# Implementation and performance evaluation of an integrated specimen referral system in Burkina Faso using the national courier services (2020–2022)

**DOI:** 10.3389/fpubh.2024.1384382

**Published:** 2024-07-30

**Authors:** Emilie Dama, Souleymane Porgho, Yahn-Cedric Ake, Issaka Yameogo, Sandrine Gampini, Aime-Gilles A. Adjami, Abdoulaye Nikiema, Mory Kamate, Felix Tarbangdo, Romial Sawadogo, Charles Sawadogo, Hamed S. Ouedraogo, Habibata Zerbo, Lila Rahalison, Isaïe Medah, Anicet G. Dahourou, Rebecca Greco-Kone, Flavien H. Ake

**Affiliations:** ^1^Division of Global Health Protection, Country Office, Center for Global Health, US Centers for Disease Control and Prevention, Ouagadougou, Burkina Faso; ^2^General Direction of Public Health, Ministry of Health and Public Hygiene, Ouagadougou, Burkina Faso; ^3^DAVYCAS International, Ouagadougou, Burkina Faso; ^4^World Health Organization, Ouagadougou, Burkina Faso; ^5^World Health Organization, Expanded Special Project for Elimination of NTDs (ESPEN), Ouagadougou, Burkina Faso; ^6^Integrated Quality Laboratory Services (IQLS), Ouagadougou, Burkina Faso; ^7^African Society for Laboratory Medicine (ASLM), Addis Ababa, Ethiopia; ^8^Directorate of Biomedical Laboratory, Ministry of Health and Public Hygiene, Ouagadougou, Burkina Faso; ^9^National Laboratory for Animal Health, Ministry of Agriculture, Animal Resources and Fisheries, Ouagadougou, Burkina Faso; ^10^Division of Global Health Protection, US Centers for Disease Control and Prevention, Atlanta, GA, United States

**Keywords:** integrated specimen referral system, Burkina Faso, laboratory system, disease surveillance, national courier services, Ministry of Health

## Abstract

**Introduction:**

In 2017, the Ministry of Health and Public Hygiene (MoH) of Burkina Faso designed and piloted a specimen transport system using the national courier services (La Poste BF) in 4 districts. Based on satisfactory performance indicators, the MoH set a vision aimed at scaling up this system to strengthen disease detection and surveillance of epidemic prone diseases across the country. This work describes the implementation process, performances, and lessons learned.

**Methodology:**

This work describes the implementation process, performances, and lessons learned. Under the leadership of the Directorate of Population Health Protection within the MoH, a stepwise approach was used to bring together multiple partners across sectors to develop the first needed documents including a guide, an implementation plan, Standard Operating Procedures, and data collection tools. Then, the execution phase included equipment purchase, trainings, and consensus on a financing mechanism. Key indicators were defined to allow performance monitoring

**Result:**

The integrated biological specimen referral system (SITEB) was officially launched in January 2020 to transport human biological specimens of priority diseases including COVID-19 from district level to reference laboratories nationwide. As of December 31, 2022, La Poste BF transported 168,856 packages containing 206,314 specimens from all 13 regions. 99.66% of packages were delivered in <24 h as required, and 99.68% of specimens were in good condition at reception. COVID-19 specimens represented respectively 18% and 63% of samples transported in 2020 and 2021.

**Discussion:**

The political will combined with the experience gained during the pilot phase and the commitment and support from all stakeholders laid to the foundation of the effective implementation of this system. Collaboration between two government entities (MoH and Minister of Transport, Urban Mobility, and Road Safety) to benefit public health has led to reasonable pricing for sustainability. Although all documents integrate the “One Health“ approach, the system ensures the transport of only human samples for now. Despite security constraints, Burkina Faso has successfully set up a system using the national postal service to ensure the routine transport of specimens for all diseases under laboratory surveillance including laboratory tests for HIV and TB from the district level to reference laboratories nationwide. This system has also proved to be useful and efficient in managing public health emergency.

## 1 Introduction

An efficient laboratory network supported by a robust transport system for biological specimens is essential to detect, prevent, and respond effectively to public health threats (1). In low-income countries, where detection capacity is particularly low in peripheral laboratories, an efficient specimen referral system is needed to support disease surveillance and the management of public health threats. To help countries achieve this, several international organizations including the World Health Organization (WHO), the U.S. Centers for Disease Control and Prevention (US-CDC), the United Nations Dangerous Goods Programme (UN DGP), and the International Organization for Standardization (ISO) provided guidance on the implementation of specimen transport systems (2–5). Despite the many guidelines and regulations, setting up an efficient specimen transport system within a laboratory network remains challenging for many countries, particularly in resource-limited sub-Saharan Africa. The main difficulties are linked to a lack of coordination, low national funding, poor implementation of laboratory policies, poor transport services, and insecurity (6, 7). Many sub-Saharan countries have been testing various means and approaches to setting up specimen transportation systems that aim to be effective and efficient despite limited resources.

Under the auspices of the U.S. President's Emergency Plan for AIDS Relief (PEPFAR) and the Global Health Security Agenda (GHSA), along with support from other international donors and NGOs, several countries have engaged in improving access to diagnostic services and surveillance systems using a performance specimens referral system. A hub network system based on different *ad-hoc* methods, including national postal courier services, was used in Uganda to increase access to Early HIV Infant Diagnosis (EID) services from 36% to 51%. This system also reduced transportation costs by 62% while reducing the turn-around times by 46.9% (8). With the support and technical assistance from a public-private partnership (PPP), the postal services were successfully used in Uganda and Ethiopia to strengthen the tuberculosis specimen referral system and increase referrals from presumptive multidrug resistant tuberculosis cases (9, 10). A similar increase in viral load tests, reagents used, and facilities accessing testing was noticed by Faruna et al. when a PPP was used to improve Nigeria's national integrated specimen referral network (11). In Malawi, earlier study conducted by the National TB Control Programme reported that peripheral units using a bus service to transport sputum to central reference laboratory for culture and sensitivity testing had a better record of specimens arriving at the CRL than those using alternative means of transport (12). While these examples have focused on transporting specimens of a specific disease, other studies have taken a more inclusive approach by integrating several diseases.

A pilot study conducted in 3 districts in Mali, included specimens from meningitis, measles, yellow fever, and polio suspected cases. This study showed that shipments of specimens from districts to the central level using the postal service was feasible and faster than public transportation. However, further analysis regarding the most efficient costing mechanism is needed (6). Inspired by the “hub” model adopted by Ethiopia and Haiti (10, 13), Guinea has developed and approved a national specimen referral policy which includes 6 diseases (Ebola, Acute flaccid paralysis, measles, yellow fever, cholera, and meningitis) using a stepwise process. The implementation of this policy has been piloted in three prefectures in Lower Guinea (14).

In 2017, a baseline assessment carried out in Burkina Faso revealed the absence of an integrated specimen transport system and highlighted the existence of fragmented disease-specific transport systems. These parallel systems were funded by different partners and used laboratory agents, increasing costs and time spent away from laboratory duties. To address this, the Ministry of Health and Public Hygiene (MoH) of Burkina Faso designed and piloted a specimen transport system using the national courier services (La Poste BF ex SONAPOST) in 4 districts under the lead of the Directorate of Population Health Protection (DPSP- Direction de la Protection de la Sante de la Population). Monitoring and evaluation of La Poste BF's performance was deemed satisfactory, with 95% of specimens sent to the reference laboratories under the appropriate conditions in <24 h and at comparatively affordable costs (15). Based on this success, the MoH has set a vision aimed at expanding and implementing an integrated biological specimen referral system, SITEB (System Intégré de Transport des échantillons Biologiques) using La Poste BF's services to transport all specimen types from districts to reference laboratories across the country. A stepwise approach was used to bring together multiple partners to develop a standardized specimen transport system that integrates other diseases and enhances laboratory capacity and public health infrastructure, thereby providing global health security implementation. This paper describes the process used to implement the SITEB using La Poste BF, the system's performance after 3 years of implementation, lessons learned, and challenges.

## 2 Methods

### 2.1 Implementation process of an integrated system for specimen transport

**Figure 1:**
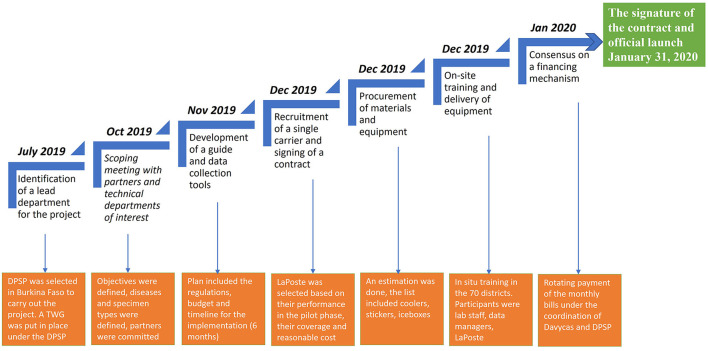
Process of the implementation of an integrated system of specimen transport in Burkina Faso. DPSP, Direction of the Protection of Health of Population (Direction de la Protection de la Sante de la Population); TWG, Technical Working Group.

Figure 1 summarizes the key stages in the process of implementing an integrated sample transport system, the chronology of their implementation and the main outcomes.

#### 2.1.1 Identification of a lead department for the project implementation and set up of a technical working group

To concretize the Ministry of Health's vision, the Directorate of Population Health Protection (DPSP-Direction de la Protection de la Sante de la Population) was designated as a lead department to collaborate closely with the Directorate of Biomedical Laboratory (DLBM-Direction des Laboratoire de Biologie Medicale) on SITEB implementation. The experience gained by the DPSP during the pilot phase with the Severe Acute Respiratory Infections (SARI) sentinel surveillance with LaPoste BF (15) was an asset for this directorate in charge of the epidemiological surveillance of diseases and also the focal point for the GHSA and the International Health Regulations (IHR) in the country.

To facilitate the project's operationalization, a SITEB technical working group (SITEB-TWG) was established and formalized by the Secretary General of the MoH. This group meets quarterly or as needed and regularly invites other stakeholders.

#### 2.1.2 Scoping meeting with partners and technical departments of interest

In low-income countries, the international partners primarily fund the transportation of specimens through several parallel systems and processes for epidemiological surveillance of most priority diseases, including meningitis, measles, influenza, dengue/arboviruses, and polio. Adopting an integrated system encompassing all specimen types across the nation necessitated the support of these partners and essential stakeholders. Some are using laboratorian technicians and other postal services or private courier services. Partners and all the MoH technical directorates involved in specimen transport were presented with the MoH's vision during this meeting. This system covers twenty-one diseases, including zoonotic, animal, and human diseases. It guarantees the transportation of around ten types of specimens from regional and health district hospitals to national and regional reference laboratories. Partners such as the US-CDC, WHO, and the African Society for Laboratory Medicine (ASLM) were represented. The key entities within the MoH involved in the specimen transport that also took part were the Directorate of Preventable Disease (DPV-Direction de la Prevention de la Maladie par la vaccination), DLBM, DPSP, and Public Health Emergency Operation Center (CORUS-Centre des Opérations de Reponse aux Urgences Sanitaires). On behalf of the Global Fund, the Health Development Support Program (PADS-Programme d'Appui au Développement Sanitaire) represented HIV and TB programs. As the MoH did not have the capacity and dedicated staff for the overall implementation of this ambitious project, an implementing partner (Davycas International) was appointed to carry out and monitor this project, including a phasing-out plan. This partner was selected based on its expertise and its capacity to work on joint projects with multiple partners and departments within the MoH to achieve public health objectives in Burkina Faso.

#### 2.1.3 Development of a guide and data collection tools

A national guide for implementing the SITEB was developed during workshops by the SITEB-TWG using a participatory, multisectoral, and multidisciplinary approach. It was then approved by the Ministry of Health during a validation workshop attended by the partners. This guide highlights the project context, the expected roles and responsibilities of the various stakeholders, and the requirements in terms of quality insurance, and biosafety and biosecurity associated with the specimen transport process. It also describes SOPs on the preparation, packaging, storage, shipping, and reception of packages, for each specimen type. Furthermore, the national guide for implementing the SITEB includes biosafety and biosecurity requirements on the category of specimens transported and international guidelines. Specific indicators were identified to ensure monitoring of the quality of the specimens transported and the overall performance of La Poste BF.

To support the implementation of this guide, job aids, and data collection tools were developed with SITEB-TWG contribution. The disease notification forms included in the SITEB have been revised to take traceability aspects into account.

#### 2.1.4 Recruitment of a single carrier and signing of a contract

Burkina Faso chose to contract La Poste BF as a courier service to transport specimens throughout the country. This semi-private courier service had collaborated successfully with the MoH during the pilot phase and was interested in this project aiming at improving the health of the population. Another important criterion was its good geographical coverage with an office in each country province. As part of implementing the SITEB using La Poste BF, the contract of the pilot phase had been revised to include other priority diseases. However, the pricing terms did not change. Same as in the pilot phase, the shipping cost was based on the weight of the coolers (2.5 kg) and the number of packages transported with and without specimens (return of empty coolers). Collaboration between two government bodies, the MoH and the Ministry of Transport, facilitated negotiations to achieve affordable pricing.

#### 2.1.5 Procurement of materials and equipment

A needs assessment was conducted based on the frequency of packages transported per week for each disease. Then, the quantity of each item was estimated, and the implementing partners placed orders. Each of the 70 districts and the eight regional health districts received three packages containing the following: plastic stickers with thermic transfer printing (humidity resistant), absorbent cotton to cushion shocks and absorb liquids in the event of spills, and an infrared thermometer to record temperature at reception. To facilitate the return of the coolers, the address of each sending laboratory was printed and attached to the coolers they received. In addition to this, the address of all other possible destination laboratories was also given to each laboratory.

#### 2.1.6 On-site training followed by delivery of equipment

The cascade training of the field agents and the handing over of the necessary equipment and support have been an important step that marked the launch of the new system. The adopted training format (region by region) gathering both field agents and those of the La Poste BF was conducted in each of the 13 regions of the country. Before the training, the SITEB-TWG developed modules covering the description of the SITEB, the role, and responsibility of the field agents, the standard operating procedures (SOPs), and related support documents. The module on the presentation of the SOPs provided details on the categorization and identification of infectious substances, the triple packaging, and the transportation and biosecurity considerations based on international resource documents such as the WHO Guidance on regulations for the transport of infectious substances 2015–2016, Laboratory Safety Manual, Third Edition, WHO 2005. In addition, guidance documents from the US-CDC and the national safety guide for medical laboratories were used. A frequently asked questions (FAQ) sheet was also developed to help trainers provide harmonized answers. Trainers were mainly SITEB-TWG members and La Poste BF agents.

To ensure the engagement and ownership of the leaders at the national level, a briefing session was organized for regional health directors and the heads of districts. This was followed by two-day training of data managers, laboratory technicians, and La Poste BF staff in all districts. People trained were clinicians, human, animal, and environmental laboratory staff and La Poste BF's transporters.

#### 2.1.7 Meeting with partners to define a financing mechanism

The partners' commitment was obtained from the start to implement SITEB, but an agreement on the financing strategy still needed to be established. Implementing a single mechanism was challenging because partners have various financial management requirements. The MoH and international partners provided the financing mechanism by establishing an annual commitment contract which includes the monthly payment schedule of the invoices. Based on the quantity of packages transported, a monthly bill is produced by La Poste BF and sent to the implementing partners and the lead department under the MoH. It was decided at the start of the year, that each partner would inform the DPSP of the number of monthly postal invoices it can handle in a year, regardless of specimen type or amount.

#### 2.1.8 The signature of the contract and launch

The signing of the agreement between La Poste BF and the MoH was followed by an official launch chaired by the MoH and attended by the regional health directors and the district chief medical officers with media coverage. Finally, a note on the implementation of SITEB signed by the MoH's General Secretary was disseminated.

### 2.2 Monitoring and evaluation mechanisms

Monitoring and evaluation (M&E) have been an essential component of the implementation of SITEB using both papers based (Table 1) and electronic data collection platform. M&E aspects have been integrated throughout the system from the case notification, specimen collection, transport, receipt, and biological results reporting. A unique labeling system with barcode stickers is assigned to each specimen to facilitate tracking.

**Table 1:** The physical data collection tools and the levels of the system where they are available and completed.

**Data collection tools**	**Description**
Record of specimen package shipments (collection sites)	In addition to data collected outside the sampling site, it collects the: - date/time of collection of the package by La Poste BF package number, - specimen or sticker number - laboratory signature - signature of La Poste BF
Examination request form (collection sites)	- Name of the prescriber - Date and time of the specimen collection - Requesting department - Examination requested. - Reason for the request and a space for the results
Individual notification form/case investigation (collection sites)	In general, the individual notification form/case investigation includes: - socio-demographic data - clinical information - sampling data - transport data and laboratory results
Summary sheet for tracking specimens (collection sites)	The summary monitoring sheets for certain specimens (sputum, specimens of animal origin) contain the name of the sampling site, a list of all the specimens contained in the cooler, and the transport data.
Package delivery form (La Poste BF/Sender, Recipient)	Issued by La Poste BF, it collects the: - date and time of collection of the package - Name of the sender - Package number and the references of the La Poste BF agent. It is signed by the senders and recipients, including the drivers, at each change of hands in order to ensure traceability.
Delivery form (La Poste BF/Sender, Recipient)	Similar to that of La Poste BF, it is used in areas where La Poste BF's services are temporarily unavailable. It is issued to the person delivering the package upon arrival.
Incident management register (La Poste BF/Sender, Recipient)	It contains the date and time of the incident; describes the type of incident (case of spillage, loss or theft of coolers, etc.), the people to contact.
Record of receipt of specimens by the laboratory/reference site. (All receiving sites)	- date and time of arrival, - the conformity of the package, - the package number, - the signature of the laboratory and La Poste BF In case of rejection of non-compliant specimens, reasons are specified

Collection sites: Health centers, Direction of preventable diseases, Direction of Animal National Laboratory, National reference laboratories.

An electronic System for Tracking of Epidemiological Data and Laboratory Specimens (STELab-System de tracabilite des donnees epidemiologiques et de laboratoire), is also used for the visualization, validation, reporting, and management of data. STELab is a web-interfaced electronic platform for case-based surveillance data entry (16). It allows the real-time recording of surveillance and laboratory data on priority diseases as well as the tracking information of a package. Its primary role was to track meningitis laboratory specimens (16). Because of the excellent results of this system, its new version has been extended to all specimens under the SITEB. Thus, today the STELab platform includes 24 diseases including zoonotic and vaccine preventable diseases. Key indicators were identified to monitor the performance of the SITEB using La Poste BF:

- *Percentage of packages delivered within 24 h*: this indicator is calculated from the time of pickup of the package from the site. The denominator is the total number of packages picked up.
- *Percentage of packages delivered in good condition:* the package at the reception does not present any non-conformity (correct packing and label).
- *Percentage of specimens delivered in good condition:* the specimens at the reception were not in good condition (Good temperature, packaging).

Data are regularly pulled from the STELab platform to produce a SITEB quarterly bulletin that is disseminated to all districts and stakeholders including partners. This bulletin summarizes the performance of La Poste BF during the reported period and since the implementation of the SITEB. The target was 100% for each of indicator.

### 2.3 Data collection and analysis

Data were collected on the STELab platform and were cross referenced with data collected by LaPoste BF. Data were confirmed, and all discrepancies were corrected. Excel software was used to develop tables and conduct all analyses presented in this manuscript.

The comparison of pre- and post- SITEB data has not been possible as there was no coordinated system in place before the implementation of the SITEB allowing centralization of data and monitoring of indicators. The performance evaluation used indicators and target percentage.

## 3 Results

### 3.1 Key outcomes from the process of SITEB implementation

The different activities implemented before the effective start of the SITEB led to key outcomes that are critical for such a system. First, there is the development of the national guide for implementing the SITEB and an operational plan of SITEB including all the SOPs. Agreement has been reached to include the following diseases in the system: Severe Acute Respiratory illness including COVID-19, dengue/arboviruses, rotavirus, norovirus, measles, tuberculosis (TB), meningitis, and Human Immuno deficiency Virus (HIV). The specimen types that SITEB can transport include Nasopharyngeal (NP) and Oropharyngeal (OP), serum, stool, sputum, pleural fluid, bronchoalveolar puncture fluid, pus, urine, and Cerebrospinal Fluid (CSF). Based on WHO guidance on regulations for transporting infectious substances (4), all selected pathogens are categorized as class 6.2 (Infectious Substances), category B. In line with this classification, the following wording has been taped to each cooler “***UN 3373, Biological substance, Category B.”***

**Figure 2:**
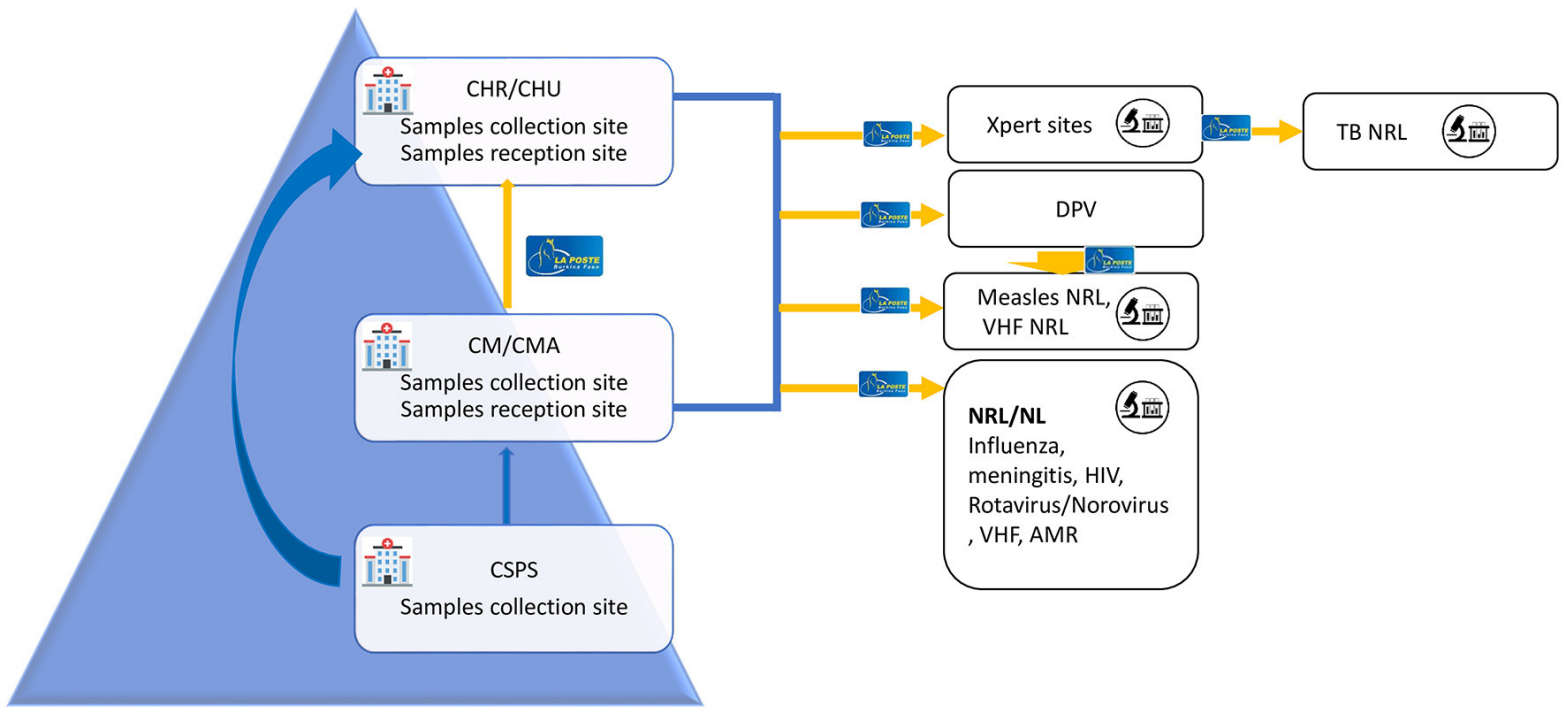
Human biological specimens transport circuit with La Poste BF Burkina Faso. CHR, Regional health facility (Centre Hospitalier Regional); CHU, university teaching hospital (Centre Hospitalier Universitaire); CMA, Medical Centre with Surgical Services (Centre Medical avec Antenne chirurgical); CM, Medical Centre (Centre Medical); CSPS, Centre for health and social advancement (Centre de Sante et de Promotion sociale); NRL, National Reference Laboratory; NL, National Level Laboratory; DVP, Directorate of vaccine-preventable diseases (Direction de la prevention des maladies evitable par la vaccination).

Figure 2 describes the specimen transport circuit in the healthcare pyramid. Specimens are transported from all the 70 districts to the national reference laboratories which include the reference laboratories for meningitis, Influenza, norovirus/rotavirus, viral hemorrhagic fevers, HIV, and TB, in addition to the immunization department that received specimens of measles and poliomyelitis.

### 3.2 Monitoring of key indicators of SITEB performance

Packages were transported from 70 districts to the national reference laboratories from all 13 country's regions. In addition to the national reference laboratories, HIV, and TB specimens were also sent to the national level laboratories since the viral load testing and TB testing are decentralized and some regions don't have the testing capacity. Ouagadougou and Bobo-Dioulasso are Burkina Faso's two largest cities, hosting all the national reference laboratories. National Reference Laboratories (NRL) for antimicrobial resistance and viral hemorrhagic fevers are in Bobo-Dioulasso and the remaining are in Ouagadougou (Influenza, meningitis, measles, rotavirus HIV, and TB).

From January 31, 2020 to December 31, 2022 La Poste BF transported 16,858 packages from the district level to the NRL and national level laboratories across the country. Among them, 99.66% (16,800/16,858) were delivered in <24 h as required in the contract with La Poste BF. Only 0.05% (9/16,856) of packages transported were found damaged during the transport. The breakdown per year shows that 14.72% (2,481) of packages were transported in 2020 against 42.30% (71,310) and 42.98% (7,246) in 2021 and 2022 respectively. No packages were reported missing or lost during transportation.

**Figure 3:**
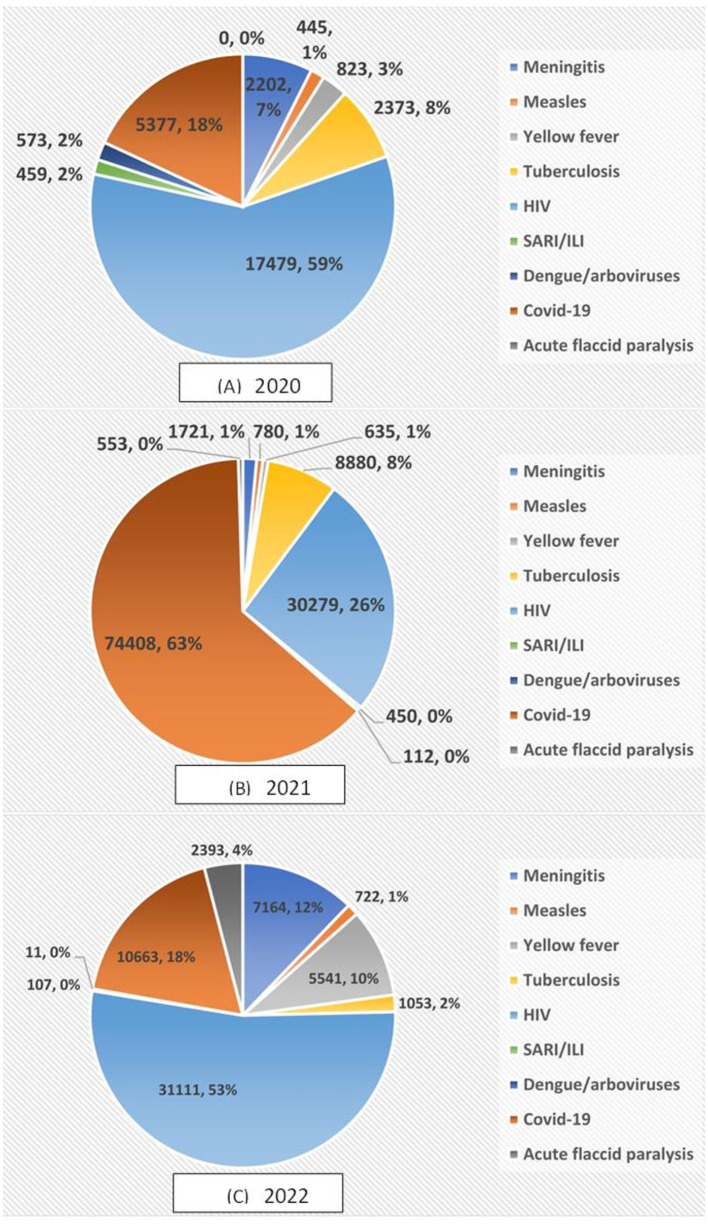
Distribution of the number of specimens transported by SITEB per disease in **(A)** 2020, **(B)** 2021, and **(C)** 2022.

During the reporting period, 206,314 specimens were transported of which 14.41% (29,731) in 2020, 57.11% (117,818) in 2021, and 28.48% (29,731) in 2022 (Table 2). The breakdown of specimens transported by disease and year reveals that in 2020 and 2022, HIV specimens were predominant with 59% and 53% respectively, while in 2021 COVID-19 specimens accounted for 63% of all specimens transported by SITEB. No specimen of acute flaccid paralysis was transported during the first year of the implementation of the SITEB whereas, in 2021 and 2022, respectively 553 (0.4%) and 2,393 (4.07%) specimens were transported (Figure 3). We didn't find any significant differences in the number of specimens transported in on year from another.

**Table 2:** Evaluation of the performance of SITEB.

**Indicators**	**2020**	**2021**	**2022**	**Total**
Packages delivered	2,481	7,131	7,246	16,858
Packages delivered in <24 h	2,437	7,121	7,242	16,800
	*98.23%*	*99.86%*	*99.94%*	99.66%
Packages delivered in good conditions	2,481	7,127	7,241	16,849
	*100%*	*99.94%*	*99.93%*	*99.95%*
Specimens delivered	29,731	117,818	58,765	206,314
Specimens delivered in good condition	29,477	117,514	58,666	205,657
	*99.15%*	*99.74%*	*99.83%*	*99.68%*

**Figure 4:**
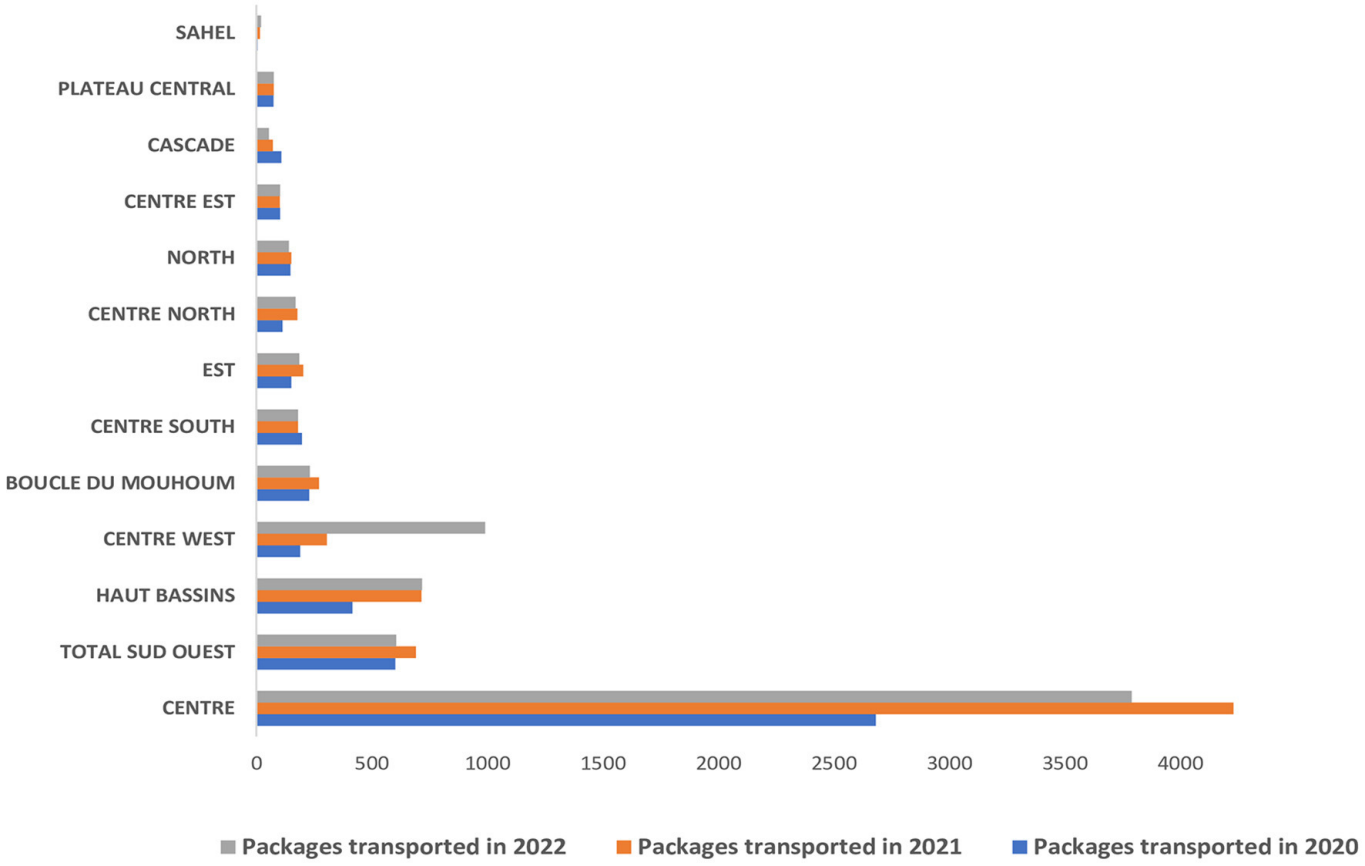
Distribution of packages transported (with and without specimens) by La Poste BF per region from 2020 to 2022.

Packages (with and without) specimens were transported from all the country's 13 regions. Overall, the number of packages transported increased between 2020 and 2022. The region with the highest number of packages is the Center region, with more than 10,000 specimens, followed by the Southwest, Hauts-Bassins, and Center West regions with more than 1,000 packages picked and delivered by La Poste BF over the reporting period. The regions where La Poste BF transported fewer packages are the Sahel (38), Plateau Central (223), Cascades (231), Center East (304), and North (348) (Figure 4).

### 3.3 Contract and rates

Like during the pilot phase, the SITEB contract was signed directly between the DPSP representing the MoH and La Poste BF representing the Ministry of Transport. The contract stipulates that La Poste BF is responsible for collecting the packages containing category B biological specimens from the public health district laboratories, delivering them to the recipient laboratory, and returning the empty triplicate packages to the sending health establishments. Two important elements of this contract are the description of the commitments of both parties and the pricing. The contract is structured on an escalating scale, with the unit price per package decreasing by 500F CFA ($ 0.84) as the number of packages to be transported increases. The minimum amount per package is 2,500 FCFA (~$ 4) and the maximum is 4,000 CFA (~$ 7) (Table 3). It is important to note that this contract is still flexible and does not provide a comprehensive list of diseases.

**Table 3:** Price list of LaPoste Burkina Faso for the transport of specimens.

**Quantity/range**	**Unit amount; TTC F CFA**	**Minimum amount; TTC F CFA (~$)**	**Maximum amount; TTC F CFA (~$)**	**Observations**
[001–500]	4 000	2,000,000 (3,229)	2,000,000 (3,229)	Fixed
[501–1,000]	3 500	2,003,500 (3,235)	3,750,000 (6,055)	Fixed price + Nb of packages ^*^ unit cost applied from the 501 st package
[1,001–1,500]	3 000	3,753,000 (6,060)	5,250,000 (8,477)	Fixed price + No. of packages ^*^ unit cost applied from the 1,001 st package
[1,501–3,000]	2 500	5,252,500 (8,480)	9,000,000 (14,531)	Fixed price + Nb of packages ^*^ unit cost applied from the 1,501 st package
[3,001 et +]	2 000	9,002,000 (14,545)	—	Fixed price + No. of packages ^*^ unit cost applied from the 3,001 st package

## 4 Discussion

SITEB is a disease non-specific system that harmonizes the transport of human biological specimens as part of national epidemiological surveillance and laboratory tests for HIV VL/EID, TB using the national postal system known as La Poste BF. To our knowledge, Burkina Faso is the first country in West Africa to implement such an integrated specimen transport system using postal services. This paper presents the stepwise process used to set up an integrated specimen transport system and its performance after 3 years of implementation. Monitoring key indicators over the 3 years of the SITEB implementation has shown the satisfactory performance of the transport of all types of human specimens from the district level throughout the country by the postal service.

This project was born of political will, followed by a clear vision of the MoH. Regulatory texts or policies must govern the specimen referral system in a country to enable effective intra- and inter-sectoral collaboration and optimization of support from partners. While some countries have developed specific policies to comply with this requirement (14), Burkina Faso, through its framework document for the development of biomedical laboratories and optimization of biological diagnosis, has clearly defined its vision about specimen transport, and listed in the same document the strategies to achieve this goal. The vision and the definition of the country's objectives in regard to specimen transport system prompted the development and validation of a national guide for the implementation of an integrated specimen transport system by the SITEB TWG based on the One Health approach.

The excellent country-wide coverage of La Poste BF's services enabled specimens to be transported to all 13 regions of Burkina Faso. However, the accessibility of some security-challenged areas due to terrorist attacks forced La Poste BF to limit its presence. This situation has led to population movements within the country, and the closure of health facilities, thereby limiting the population's access to healthcare (17, 18). In Burkina Faso, the regions most affected by the humanitarian crisis are the Sahel, Center-North, Nord, Est, and Boucle du Mouhoun. Although not on a continuous and systematic basis, La Poste BF has been transporting specimens from functional districts in these security-challenged regions since the launch of SITEB. When this proved impossible for security reasons, other strategies were developed and deployed. An additional factor to explain the considerable diversity in the number of packages per region is the COVID-19 crisis. More than half of the packages transported came from the central region, which was the epicenter of COVID-19, followed by Hauts-Bassins region with 15,712 and 3,517 cases detected between 2020 and 2022 representing more than 70% and 15% of the total cases respectively (19).

From the launch of SITEB in January 2020 to December 2022, out of 16,858 packages transported, 99.66% were delivered within 24 h from pick-up time at the collection site (the required turnaround time), compared to 77% during the pilot phase (15). This result shows a significant improvement in the post office's performance, dispelling initial fears about its ability to meet this challenge. To carry out its mission by the agreed upon contract, La Poste BF has signed an agreement with several public transport companies in the country's main cities, hired additional staff and procured logistical resources. A similar pilot study in Mali showed that only 46% of specimens transported by public transport system were delivered within the required timeframe (72 h), compared to 71% of specimens transported by Mali's postal service specific means of transportation. The same study found a comparable percentage of specimens delivered in good conditions between the two types of transport (6). Indeed, the public transport network in Burkina Faso is diversified and well organized, with regular departures to major cities. While transporting biological specimens in public transport vehicles can be perceived as a risk, the triple packaging and extra protection provided by La Poste BF help to further minimize the risk of exposure to potentially dangerous pathogens contained in the specimens. Using drones or unmanned aircraft System technology is being explored by some studies to transport specimens, vaccines, and other laboratory supplies. However, a cost-effectiveness analysis of the use of these new technologies which integrates all considerations (including security) must be conducted (20–22).

The flexibility of the contract provisions to permit the integration of additional specimens or adjustments during their term is one of the system's features and success. In 2021, this encouraged the incorporation of COVID-19 and acute flaccid paralysis specimens. COVID-19 had not yet been declared a pandemic by the World Health Organization (WHO) at the time of the SITEB's launch, and the country intended to keep using the traditional system for transporting acute flaccid paralysis specimens because the disease was considered on its way out. During the 1^st^ months of COVID-19 in Bobo-Dioulasso, there was only one laboratory in the country capable of performing diagnosis and it was in Bobo Dioulasso. MoH vehicles transported specimens from suspected COVID-19 in other regions to Bobo-Dioulasso. However, as the number of cases increased across the country, specimen transportation became difficult due to logistical issues. COVID-19 specimens were integrated into the SITEB without any changes to the initial contract or pricing after several meetings and briefing sessions with the post office.

There was little to no significant difference in the number of packages that La Poste BF transported in 2021 and 2022. However, the number of specimens transported was twice higher in 2021 due to COVID-19 specimens (63%) which were transported by the dozen in a single package. Indeed, the peak of COVID-19 cases was notified in 2021 between January and February with more than 69,000 COVID-19 samples tested. It is worth noting that this number includes those of COVID-19 suspected cases but also samples collected from international travelers who are required to test. The drop in COVID-19 cases and the implementation of vaccination in June 2021 explains the decrease in COVID-19 specimens collected in 2022. The decrease in the number of specimens transported by SITEB for other diseases such as meningitis, yellow fever, SARI/ILI, and dengue/arbovirus between 2020 and 2021 can be attributed to the impact of COVID-19 on the health system in general and on disease surveillance in particular, as documented in numerous studies (22, 23). Several initiatives and actions were implemented to re-energize disease surveillance, which had been slowed by COVID-19, and improvements were seen in late 2021 and 2022.

In most developing countries, disease surveillance, including specimen transport, is funded by international partners. To minimize the risk of this system collapsing due to a lack of resources, particular emphasis was placed on negotiating rates. Rates were negotiated between the Ministry of Health and La Poste BF to ensure that the country would be able to meet costs in the event of a reduction or cessation of partner support. During the pilot phase, which only involved 4 districts, the cost of transporting a package by La Poste BF was around 28 USD (17,500 CFA), when the system was extended, the cost was negotiated to ~6 USD (3,500 CFA) per package, almost 5 times cheaper. The estimated cost for implementation of the SITEB in Burkina Faso is approximately 662,000 USD (400.000.000 CFA) which includes meetings, equipment, trainings, and document printing. The average cost of the LaPoste BF's monthly bill is ~23,528 USD (14.215.762 CFA). The cost-benefit analysis of such a system using postal services or hub systems is still a gap in many studies (23).

## 5 Lessons learned

The experience of Burkina Faso provides important lessons and recommendations that must be considered to ensure the successful development and implementation of an efficient and integrated specimen transport system. The following are key lessons learned from this experience:

- A strong political will is essential to engage partners and stakeholders.
- It is critical to select an efficient operator (public or private) capable of providing services throughout the country.
- Contract flexibility is essential so that, in addition to surveillance and clinical diagnosis, the system can be used in response to epidemics or other public health events.
- Throughout the process, sustainability, and a multi-sector approach (One Health) must be considered.
- A good monitoring and evaluation plan must be developed to ensure that the system runs smoothly and to allow assessing performance and impact of the system.
- To ensure specimen transport in insecure areas where government offices and health facilities are not operational, an innovative strategy must be developed.
- It is important to consider an implementation and coordination partner with dedicated staff to ensure smooth implementation while ensuring a phase-out.

## 6 Challenges and perspectives

The main limitation of the SITEB is the non-integration of animal and environment specimens in this system. Although, the guide and all data collection tools have been revised according to the One Health approach, the implementation must still be effective. Discussions are ongoing to make this happen. In the clauses of the current contract La Poste BF picks up specimens from the district level while there is no formal system in place to transfer specimens from peripheral level to district level. A pilot phase is underway in 2 regions where La Poste BF picks specimens from the peripheral level to extend it to the entire country after an evaluation and a revision of the contract. Finally, although the partners have put in place a mechanism for paying monthly postal bills, the SITEB's operating costs (SITEB-TWG meetings, supervision, equipment replacement, document printing) still need to be included, and there needs to be a government budget line to support the operation of this system.

## 7 Conclusion

The involvement of stakeholders at all levels, as well as partners, contributed to the success of this innovative system. Furthermore, the success and lessons learned from the pilot phase (15) have made a significant contribution to laying the foundations of this integrated system, which is now widely used in the surveillance of priority diseases in Burkina Faso, as well as in the management of health crises. Several countries have attempted to use national mail services to transport biological specimens to strengthen surveillance of a country's set of priority diseases or specific diseases such as tuberculosis and/or HIV (10). However, Burkina Faso is one of the countries that has successfully implemented a national mail service for a specimen transport system, which considers all the diseases under laboratory-based surveillance and covers the whole country down to the district level. The performance of the SITEB after 3 years of implementation made it a major pillar in laboratory-based surveillance of priority diseases in Burkina Faso. It ensures the transport of all specimens collected for surveillance purposes including VIH and TB from district level across the country. Thanks to its flexibility, it also plays an important role in the management of public health emergencies for an early detection and quick response. The integration of animal specimens remains a big gap, but efforts are underway to address this.

## Data availability statement

The raw data supporting the conclusions of this article will be made available by the authors, without undue reservation.

## Author contributions

ED: Conceptualization, Data curation, Formal analysis, Funding acquisition, Methodology, Project administration, Resources, Validation, Visualization, Writing – original draft, Writing – review & editing. SP: Conceptualization, Data curation, Formal analysis, Methodology, Supervision, Visualization, Writing – review & editing. Y-CA: Data curation, Formal analysis, Methodology, Visualization, Writing – review & editing. IY: Conceptualization, Methodology, Project administration, Supervision, Writing – review & editing. SG: Funding acquisition, Methodology, Resources, Writing – review & editing. A-GA: Conceptualization, Methodology, Supervision, Writing – review & editing. AN: Conceptualization, Funding acquisition, Methodology, Writing – review & editing. MK: Data curation, Methodology, Visualization, Writing – review & editing. FT: Data curation, Methodology, Visualization, Writing – review & editing. RS: Methodology, Validation, Writing – review & editing. CS: Funding acquisition, Methodology, Resources, Writing – review & editing. HO: Resources, Supervision, Validation, Writing – review & editing. HZ: Methodology, Validation, Writing – review & editing. LR: Funding acquisition, Methodology, Writing – review & editing. IM: Conceptualization, Methodology, Writing – review & editing. AD: Conceptualization, Methodology, Validation, Writing – review & editing. RG-K: Conceptualization, Funding acquisition, Project administration, Supervision, Writing – review & editing. FA: Conceptualization, Data curation, Funding acquisition, Methodology, Project administration, Resources, Supervision, Validation, Visualization, Writing – review & editing.
